# State Recognition of Bone Drilling Based on Acoustic Emission in Pedicle Screw Operation

**DOI:** 10.3390/s18051484

**Published:** 2018-05-09

**Authors:** Fengqing Guan, Yu Sun, Xiaozhi Qi, Ying Hu, Gang Yu, Jianwei Zhang

**Affiliations:** 1 Shenzhen Institutes of Advanced Technology, Chinese Academy of Sciences, Shenzhen 518055, China; fq.guan@siat.ac.cn (F.G.); yu.sun@siat.ac.cn (Y.S.); 2 Harbin Institute of Technology at Shenzhen, Shenzhen 518052, China; gangyu@hit.edu.cn; 3 Department of Informatics, University of Hamburg, 22527 Hamburg, Germany; zhang@informatik.uni-hamburg.de

**Keywords:** pedicle drilling, acoustic emission signal, frequency distribution, neural network

## Abstract

Pedicle drilling is an important step in pedicle screw fixation and the most significant challenge in this operation is how to determine a key point in the transition region between cancellous and inner cortical bone. The purpose of this paper is to find a method to achieve the recognition for the key point. After acquiring acoustic emission (AE) signals during the drilling process, this paper proposed a novel frequency distribution-based algorithm (FDB) to analyze the AE signals in the frequency domain after certain processes. Then we select a specific frequency domain of the signal for standard operations and choose a fitting function to fit the obtained sequence. Characters of the fitting function are extracted as outputs for identification of different bone layers. The results, which are obtained by detecting force signal and direct measurement, are given in the paper. Compared with the results above, the results obtained by AE signals are distinguishable for different bone layers and are more accurate and precise. The results of the algorithm are trained and identified by a neural network and the recognition rate reaches 84.2%. The proposed method is proved to be efficient and can be used for bone layer identification in pedicle screw fixation.

## 1. Introduction

At present, pedicle screw fixation, which is the most popular technique in spinal fixation surgery [1], is often used in vertebral dysfunction caused by spinal deformity, injury or pathology. In the screw fixation process, screw drilling is one of the most critical steps. There are also many organs, tissues and nerves distributed around the spine [2]. To avoid the irreversible damages to them, and meanwhile ensure good stiffness as well as biomechanical properties of the spine [3,4], depth of the drilling bit into bone layers has to be accurately controlled in the operation while the angle between the drilling bit and the bone must be well chosen at the beginning of the process [5]. Traditional spine surgery mainly depends on surgeons’ experience and skills in performing the surgery, so tiny mistakes of a doctor are likely to cause failure of the surgery.

During the drilling process, the invisibility of the osseous structure, along with consistent and stable friction between the pedicle and the cutting edge of the tool, makes it hard to directly measure the penetration depth. Therefore, it’s only possible to acquire different types of signals during the drilling process and extract features to estimate the depth of penetration. Signals which can be used for analysis are mainly image signals, force/torque signals, current signals and sound signals.

Systems using image signals for navigation can significantly improve accuracy of pedicle screw insertion and allow a surgeon to evaluate screw trajectory by using preoperative or intraoperative acquired images at the same time [6,7,8]. However, lots of existing devices and methods rely on force and torque transducers to measure thrust forces and torques, and then detect bone layers and changes between the different bone layers from the detected signals. Online monitoring of the state of bone drilling is essential to improve operational safety and the surgeons’ operating skills. Based on the estimated cutting resistance that was detected by a motion sensor, the hand-held bone cutting system, which was proposed by Osa et al., could automatically stop moving and avoid the penetration of inner cortical bone [9]. Lee et al. proposed a force controller to automatically stop at critical points. In addition, Hu et al. and Mohd et al. proposed a real-time force sensing algorithm based on force signals to identify different bone layers [10,11,12]. By measuring force signals, Kaburlasos et al. estimated tarsal thickness in the surgery [13] and Federspil et al. developed a force controlled robot to detect the dura in neurosurgery [14]. Also, Kim et al. proposed a force feedback scheme to reduce the force exerted by positioning robot and provide force feedback to the surgeon through a dual moment sensor in long bone fracture operation [15].

However, the force signal is noisy and filtering introduces a certain delay. The delay is determined by the filtering method and the equipment they used. Also, determination of the bone layers by force signals is influenced by the threshold value used. A high threshold will cause the identification to be too late while a low threshold will lead to false detections. Accini et al. proposed a novel algorithm to detect position of the drilling bit and realize the control of bone drilling only based on a position sensor [16]. Dai et al. pointed out that bone status can be monitored by analyzing the vibration signals of bones and presented a non-contact system to collect and analyze the vibration signals by using a laser displacement sensor, and eventually the system achieved real-time detection of the drilling state in thoracic surgery, overcoming the shortcomings of any kinds of contact sensor [17].

Some people have gradually turned their attention to the study of acoustic emission (AE) signals. AE signals are well studied and applied in the classification of composite material damage mechanisms [18,19], tool wear analysis [20,21] and detection of crack damage based on ultrasonic testing [22]. However, in the aspect of identification and recognition of bone layers, the AE signals are still in a research phase. Boesnach et al. analyzed the AE signals in the process of spinal drilling and proposed that sound signals have a strong correlation with bone mineral density [23]. Pohl et al. used a sound sensor to collect the AE signals to detect the state of penetrating of mice skulls [24], and the bone layer was successfully identified. In 2014, Sun et al. analyzed the AE signals collected during drilling process through Fast Fourier Transform (FFT) and used Exponential Mean Amplitude and Hurst Exponent to verify energy characteristics and stabilities of the AE signals, and then developed a real-time algorithm to identify the bone layers by detecting the AE signals [25]. Liao et al. analyzed histological structure and mechanical properties of the bone layers and studied the relationships between the AE signals and the aspects including formation of chips, depth of penetration and cutting faults in the drilling process. They declared that the AE signals contain much useful information and have good potential for studies and applications [26].

The purpose of this study is to explore an innovative method of monitoring key points based on AE signals during pedicle screw drilling. Firstly, the AE signals in the bone drilling process are collected by a sound sensor and preprocessed by a recursive fast Fourier transformation (FFT), and then the preprocessing results will be further processed by a frequency distribution-based algorithm (FDB). The coefficients obtained from the FDB algorithm will be used in characterizing the shapes of fitting functions, which can indirectly characterize the recognition of the bone layers. Finally, a neural network is used to train and test to verify accuracy of the proposed method.

The structure of this paper is organized as follows: Section 2 details bone layer analysis and AE signal acquisition. Section 3 presents the proposed FDB algorithm in detail and briefly introduces the structure and parameters of the neural network. Experiments are carried out and the results are analyzed in Section 4, then conclusions are given in Section 5.

## 2. AE Signal Acquisition in Bone Drilling

### 2.1. Structural and Mechanical Properties of Bone

A typical bone structure consists of cortical and cancellous bone, which have different mechanical properties [27]. In general, cortical bone, which has good mechanical properties, can be considered as a reinforced composite material. The cortical bone plays an important role in supporting human body as well as protection of organs. Meanwhile, cancellous bone, which has low density and elastic properties, and uniformly fills the inner area of the cortical layer [28], is a soft tissue and helps to maintain skeletal form and resist pressures coming from outside.

**Figure 1:**
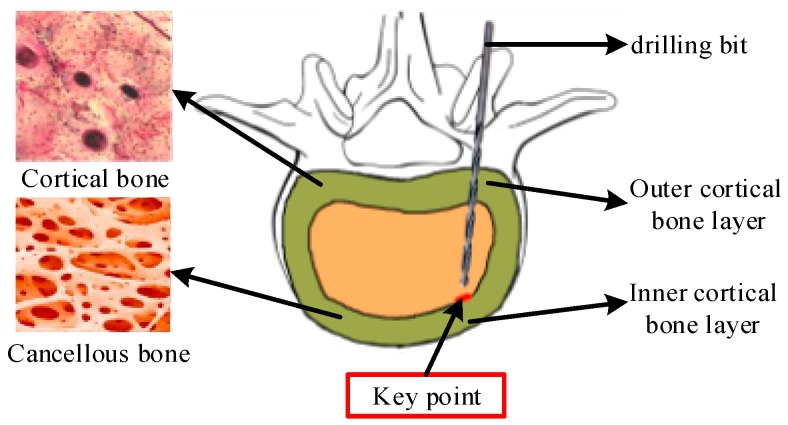
The structure of single segment in pedicle and the bone drilling in the spine pedicle, where light green represents the cortical bone and canary yellow represents the cancellous bone.

As shown in Figure 1, when drilling a path in a pedicle, the drilling bit drills the targeted bone from outer cortical bone layer, goes through middle of cancellous bone and then stops in front of the inner cortical bone. In this way, any weakening of the mechanical properties of the spine can be minimized. Therefore, it is particularly important to detect the key point and for the drilling bit to then stop in front of the inner cortical bone. The research described in this paper aims to extract the features of AE signals during the process and realize the identification of the key point after subsequent processing.

### 2.2. Signal Acquisition System

The system that collects AE signals mainly consists of two parts: an AE signal detector sensor and a NI data acquisition card. The AE signal detector sensor, produced by the Spark Fun Company (Boulder County, CO, USA), can be used for acquisition of AE signals during the drilling process. The AE signal detector is a small board that combines a microphone and some processing circuitry. It provides not only an audio output, but also a binary indication of the presence of sound, and an analog representation of its amplitude. With its three separate outputs, the board itself is a lot more flexible and it is easy to see what each is doing with a graph. The output pin we choose in the experiments is the audio output. The power supply voltage ranges between 3.5 and 5.5 V and 5 V is an ideal value. The signal acquired by the AE detector sensor is an analog signal, which is continuously distributed at any time interval. In order to sample and study the digital signal in detail, an acquisition card is needed to discretize the analog signals. The NI data acquisition card is a USB-6002, produced by National Instruments (Austin, TX, USA). The analog-to-digital converter resolution is 16 bits and the maximum sampling rate is 50 kHz. The main function of the data acquisition card is to automatically measure voltage signal input from the AE signal detector sensor, and then send the data to a host computer for analysis.

**Figure 2:**
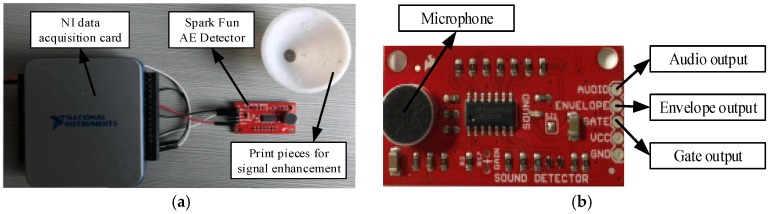
(**a**) The AE signal acquisition system, where (**b**) is the AE detector used for AE signal collection, the NI data acquisition card is used to send the data to computer and the printed piece is used for signal enhancement.

In order to enhance the intensity of the acquired AE signals and reduce the ambient noise interferencef, a 3D printed device, which is the print piece for signal enhancement in Figure 2 and has the conical surface, is mounted on the front end of the microphone module. The conical surface of the device allows the device to reduce the spread and the attenuation of the AE signals. A specific device diagram is shown in Figure 2.

### 2.3. AE Signal Acquisition

**Figure 3:**
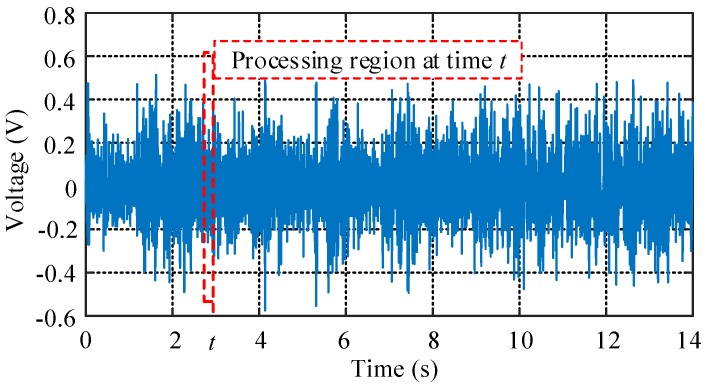
The AE signal during bone drilling in time domain, part of the signal in the dashed frame is used for subsequent signal processing of time *t*.

The AE signals during the drilling process are generated by friction between the drilling bit and the bone layer. In order to extract a characteristic amount of the AE signal and perform subsequent processing, the generated signal needs to be collected. During the experiment, the above experimental device is used to collect the signal. The sampling frequency is set to 44,000 Hz, according to the Shannon sampling theorem [22], frequencies below 22,000 Hz can be collected. The maximum sampling frequency of the data acquisition card is 50 kHz, therefore, it fully meets the conditions of use. In a complete collection process, the force signals are also recorded as a reference at the same time. When the drilling bit is idling, the amplitude of the sound signal is small. The moment the drilling bit hits the bone, the signal amplitude increases dramatically. The moment the drilling bit drills through the bone, the amplitude of the signal decreases significantly. Based on this, we can select a period of sound signal from drilling into the outer cortical bone to drilling through the inner cortical bone, and the selected signal in the time domain during the process is shown in Figure 3.

It can be seen from Figure 3 that the volume of the AE signal is very large and the distribution of the data is quite intensive, so the differences between various bone layers can’t been distinguished easily in the time domain by simple methods. Therefore, the original signal needs to be processed to obtain certain characteristics for achieving the recognition of different bone layers.

## 3. The Algorithm for the State Recognition

### 3.1. Pre-Processing with FFT

The characteristics of the AE signal in the time domain are not intuitive. To get more information, the signal needs to be transformed into the frequency domain for analysis. In order to realize real-time identification of the bone layers, it is necessary to analyze the frequency-domain features at each moment. Fast Fourier Transform (FFT) is an ideal way to achieve this.

**Figure 4:**
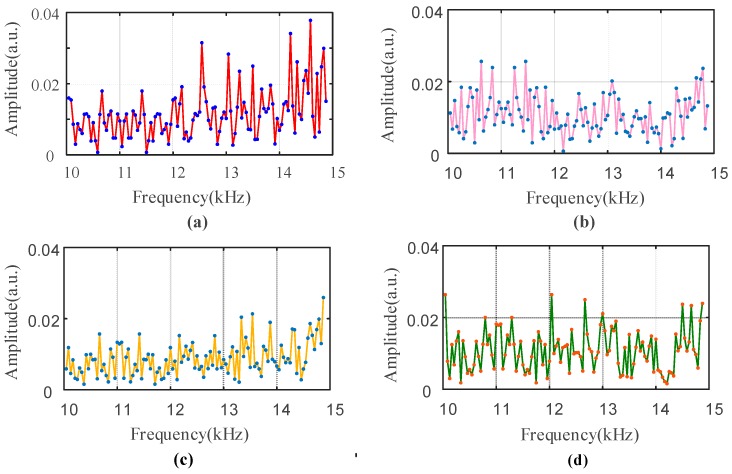
The distribution of the frequency between 10 kHz and 15 kHz after using the recursive FFT for different moments, where (**a**) corresponds to the moments of drilling the cortical bone; (**b**) is related to the cancellous bone; (**c**) is the transition region from cortical bone to cancellous bone and (**d**) is the transition region from cancellous bone to the inner cortical bone. In addition, the *y*-label is energy intensity distribution at various frequency values.

Since the FFT reflects global features that can’t represent the spectral characteristics of features at each moment, the signal is processed by a recursive FFT. To reduce or eliminate spectral energy leakage and fence effects, different interception functions, also called window functions, can be used to truncate the signals. We choose a Hamming window with a frame size of 512 data points and a frame shift with 100 data points. The chosen area, which is denoted by a red dotted box and contains a certain number of data points in Figure 3, can be considered as the original data for time *t*, which is a moment randomly chosen for further analysis. By referring to the conclusions, which were obtained from many sets of experiments we conducted, the frequency of the drilling bone process is mainly distributed in range of 10 kHz and 15 kHz. The signals in the frequency of this range are less affected by noise interference for the reason that normal noise are not distributed in this frequency range. Further study of the signals in the selected frequency confirms it can well represent the drilling process. By referring to force signals collected simultaneously, we chose the moments of the signals for different bone layers and use the recursive FFT to process them. The frequency distributions of the chosen moments are shown in Figure 4.

By comparing the distribution for amplitudes in Figure 4a–d, it can been seen that the amplitude distribution for cortical bone layer is concentrated in high frequency bands while the distribution for cancellous bone layer is concentrated in lower frequency bands. However, compared with the former graphs, the amplitude distribution of the transition region is relatively uniform for the reason that the local maxima are smaller than the values in the former graphs and the distributions have little differences during the changes of values. In another words, the distribution can also be considered as be concentrated in intermediate frequencies for the transition region. This conclusion can be further analyzed and some certain characteristics can be found to describe the regularities.

### 3.2. Analysis of the Energy in Bone Layer

Different bone components have different sound transmissibility, and cortical bone is denser, so it can form a better reflection even for shorter wavelength signal components, so the transmittance is poor. Cancellous bone has a loose porous structure, so high-frequency components can be diffused within the bone through these pores and be attenuated by vibration to achieve better transmittance, so a medium with low transmittance will reflect more high frequency components, which include greater energy and can be collected by the sensor. Meanwhile, the medium with better transmittance will reflect a small amount of any high-frequency components, so the high-frequency energy received by the sensor is much smaller than the former.

For any moment *t*, we select a fixed number of sampling points that include the current moment for the FFT transformation to obtain a frequency spectrum at this moment. The frequencies in the spectrum are distributed between 0–22,000 Hz. The above analysis shows that the cortical bone contains more high-frequency energy and the cancellous bone contains more low-frequency energy, while the energy is evenly distributed in the transitional region. Then we select s frequency between 10 kHz and 15 kHz from the spectrum signal, so *k* pieces of data corresponding to moment *t* can be obtained, that is:

(1) $$F_{i}={\left(x_{1i}\;x_{2i}\;\ldots \;x_{ki}\right)}^{T}$$

where *x_ki_* are values of the amplitude and **F***_i_* is the whole sequence of the moment *i*. The energy of bone layers is proportional to the sum of square of the amplitudes.

### 3.3. Algorithm Based on Frequency Distribution

When drilling different layers of bone, the energy in different frequency bands will have different distributions, which means a large difference in the energy value of the signal in the selected frequency range. The most intuitive feature at this point is that the curve composed of **F***_i_* differs in the frequency domain and the shape of the curve varies when the layers of the bone are different. By describing the shapes of various curves, it is possible to show the characteristics of the curve to a certain extent and then reflect the energy distribution.

#### 3.3.1. Normalization and Sorting

In order to eliminate the influence of the energy factor in the time domain and to unify different data on the same scale for subsequent analysis and processing, the number of *k* data obtained above is normalized by Equation (2):

(2) $$F_{ni}=\frac{F_{i}}{\max(F_{i})}$$

where **F***_i_* is the chosen sequence and max (**F***_i_*) is the largest value of **F***_i_*, the result **F***_ni_* is a new sequence after normalization. To describe the sequence more precisely, a method that sorts the sequence **F***_ni_* from small to large in a certain order is discovered in Equation (3):

(3) $$U_{ni}=sort\;(F_{ni})$$

**Figure 5:**
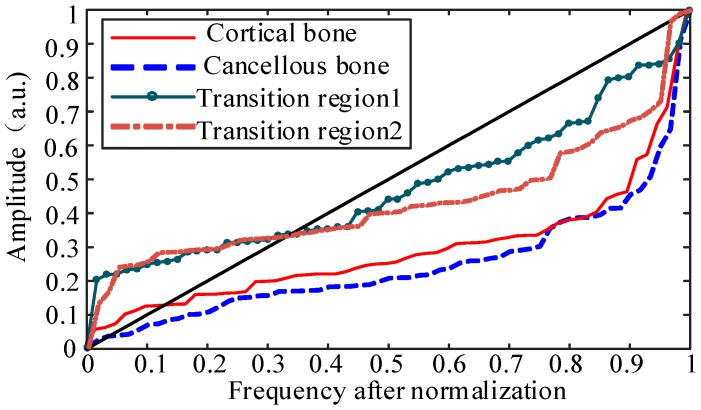
The relative distribution between amplitude and the frequency after normalization and sorting for the chosen frequency bands in different bone layers.

The result **U***_ni_* is an ascending curve in the unit domain and it can continuously reflect the relative distribution of data at various energy levels. After using the proposed method, the curves in Figure 4 can be transformed separately into the curves in Figure 5.

The curve corresponding to *y* = *x*, which is also the diagonal of the figure, is defined as an “original line”. When the energy distribution in the drilling process is relatively uniform, that is, no large transmission occurs, the distribution of energy in all frequency bands tends to be uniform as a whole. The ascending curve is distributed near the “original line”, which corresponds to the curve indicated by the dark green solid line with dots and the brown dot line in Figure 5.

When the energy distribution in the process of drilling bone is concentrated in some certain frequency bands, after normalization and sorting processing, the curve deviates from the “original line”. However, the function curve can’t distinguish the signals of high-frequency energy bands from low-frequency energy bands. The reason is that when the energy is concentrated in low-frequency bands, after processing, the role of the low-frequency energy segment is more significant and will be concentrated in the latter part of the curve, which is the same as the situation when the energy is mainly distributed in high frequency bands. Just as the Figure 5 shows, it’s hard to distinguish the red solid line which represents the cortical bone from the blue dotted line that represents the cancellous bone.

However, the main objective of this study is to identify the key points in transitional regions between cancellous and inner cortical bone. Compared with cortical and cancellous parts, the energy distribution in transitional regions is relatively uniform, so the transition area can be distinguished from the whole drilling process. However, there is still a problem to be solved. A function, whose coefficients can represent the characteristics of the curve and identification of the bone layer, is needed to fit the data.

#### 3.3.2. Coordinate System Conversion

**Figure 6:**
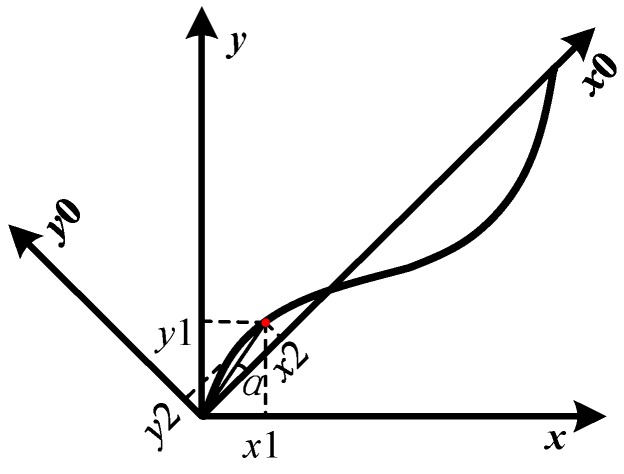
The conversion between coordinate **C**1 and **C**2.

By studying a number of experimental data, it’s found that the ascending curve is distributed on both sides of the straight line *y = x* in coordinate **C**1, and the corresponding different layers of the bone exhibit a certain regularity. However, it is difficult to fit the curve in Figure 5. It is possible to transform the curves distributed on both sides of *y = x* to the distribution along the *x*-axis by coordinate system conversion, which means the curve in *xoy* coordinate system **C**1 can be transformed into *x*_0_*oy*_0_ coordinate system **C**2 for analysis, just as Figure 6 indicates.

For any point (*x*_1_, *y*_1_) on the curve, the relationship between the two coordinate systems can be established using Equation (4):

(4) $$\left\{ \begin{array}{l} x_{1}=\sqrt{x_{2}{}^{2}+y_{2}{}^{2}}\cos(\alpha +\frac{\pi }{4})\\ y_{1}=\sqrt{x_{2}{}^{2}+y_{2}{}^{2}}\sin(\alpha +\frac{\pi }{4})\\ \alpha =\arctan\frac{y_{2}}{x_{2}} \end{array}\right.$$

The solutions of Equation (5) are:

(5) $$\left(\begin{array}{l} x_{2}\\ y_{2} \end{array}\right)=\left(\begin{array}{cc} \frac{\sqrt{2}}{2} & \frac{\sqrt{2}}{2}\\ -\frac{\sqrt{2}}{2} & \frac{\sqrt{2}}{2} \end{array}\right)\left(\begin{array}{l} x_{1}\\ y_{1} \end{array}\right)$$

so the relationship between the coordinate **C**1 and **C**2 is:

(6) C2=A⋅C1

where **A** = $\left(\frac{\sqrt{2}}{2}\;\frac{\sqrt{2}}{2};-\frac{\sqrt{2}}{2}\;\frac{\sqrt{2}}{2}\right)$ is the transformation matrix between the two coordinates **C**1 and **C**2. 

**Figure 7:**
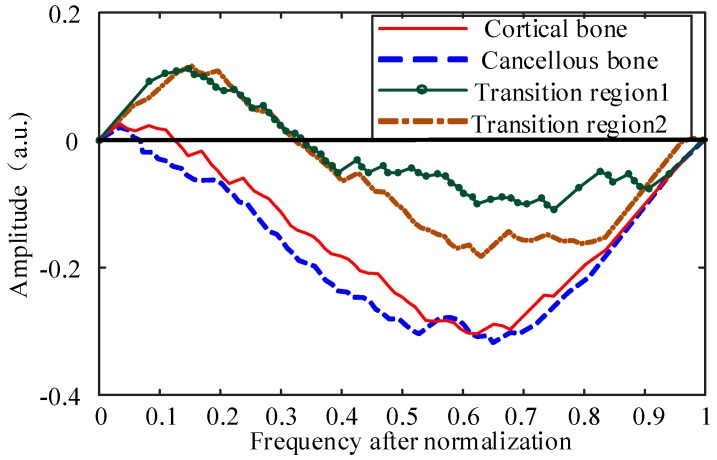
The new curve representing the relationship between the amplitude and the frequency after conversion and normalization for different bone layers. Each of the curves corresponds to the curves shown in Figure 5.

By using Equation (6), the ascending curve representing the relationship between the amplitude and the frequency in Figure 5 can be converted into a new curve, just as Figure 7 shows.

#### 3.3.3. Fitting Function Selection and Evaluation

As can be seen in Figure 7, the curve is distributed on both sides of *x*-axis and appears to be in the shape of a sine curve. Therefore, the fitting function should include a sine term that is across the points (0, 0) and (1, 0) and try to keep the function in a sine cycle. In addition, the change of coefficient of the sine function should also be able to control the intersection of curve and *x*-axis, so the sine function can be expressed in the form:

(7) $$y_{1}=\sin[(2\pi +cx(1-x))\cdot x]$$

where *c* is the coefficient to control the location of the intersection point.

At a different time *t*, the amplitude of the function curve will change accordingly. In addition, the amplitude of the curve increases when the value of *x* increases, which is more obvious especially when the value of *x* is large enough to make the curve be in the lower half of the *x*-axis, so the amplitude of the function should also be related to the argument *x*. An exponential relationship can be found between the variable *x* and the amplitude. Thus the fitting function also has an item in form *ae^bx^*. Then the most intuitive performance is that the amplitude ratio between the amplitude below and above the *x*-axis is related with the coefficient *b*. To sum up, the function of the form (8) has a good fitting effect:

(8) $$y=a\cdot e^{bx}\cdot \sin[(2\pi +cx(1-x))x^{d}]$$

where *a*, *b*, *c* and *d* are coefficients of the fitting function, which are determined by the original data and fitting function together. The coefficients will be used to represent the identifications of different bone layers.

**Figure 8:**
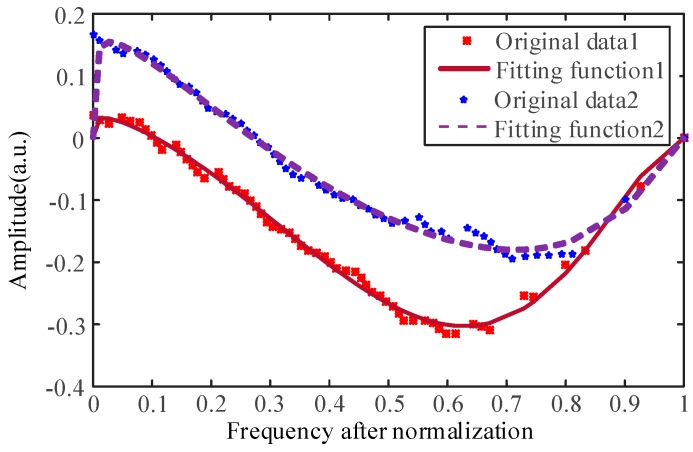
The original data after sorting and the curve of the fitting functions, where the points represent the original data and the curves are the fitting functions.

We selected two groups of data corresponding to different moments which represent the cortical bone layer and transition region for fitting and then evaluated the fitting process. The curves are shown in Figure 8, and the results of the judging process and evaluation results are shown in Table 1.

In Table 1, SSE is the sum of squares due to error, so the closer the SSE is to zero, the better the effect of model selection and fitting is, and the more successful the data prediction is. R-square is the coefficient of determination and is used to characterize the goodness of fit and normality. The range of values is between 0 and 1, and the closer it is to 1, the better the model at fitting the data. RMSE is the root mean square error, which is also called fitting standard error of the regression system. It is the square root of the mean of squares of point errors of the prediction data and the original data. The smaller the value is, the better the fitting function is. It can be seen from the data in Table 1 that the chosen fitting function has a good fitting effect and strong ability to explain the original curve. Therefore, the coefficient of the fitting function can well characterize the changes of the curve.

#### 3.3.4. Analysis of the Algorithm’s Coefficients

The coefficients of the fitting function can well characterize the changes of the curve. When the drilling bit goes through the cortical bone and cancellous bone, the energy is more concentrated. After normalizing and sorting, the distribution of the latter part of the upward curve is more concentrated, while the distribution of the former part is sparser. Then for the rising curve, the number of the data above the “original line” is quite small. After the conversion of the coordinate system, the number of data above the *x*-axis is small, the most intuitive performance of which is that the intersection of the curve and *x*-axis is distributed in the front of the interval [0, 1], and the amplitude above the *x*-axis is small while the amplitude ratio mentioned above is quite large.

When the drilling bit is in the transitional region, the energy is distributed in the high-frequency and the low-frequency part at the same time. After processing, the distribution is scattered and relatively uniform around the “original line”, leading to the phenomenon that the intersection of the curve and *x*-axis is distributed in the middle part of the interval [0, 1] and the amplitude above ***x***-axis is larger while the amplitude ratio is smaller.

**Figure 9:**
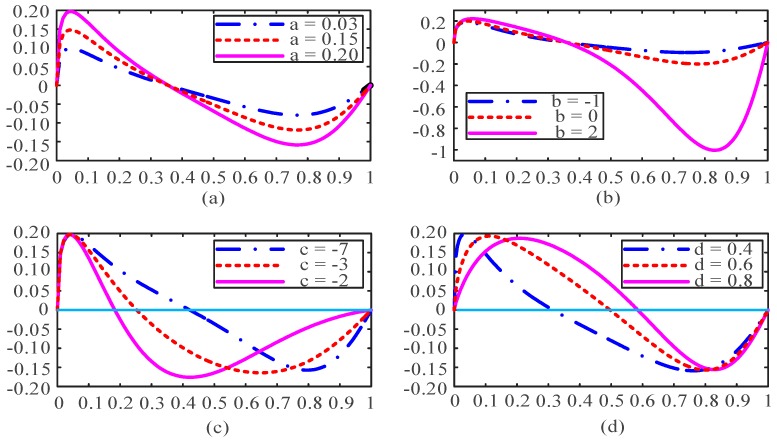
Changes in shape of the curve under the influence of coefficients, where (**a**) shows different curves when the coefficient *a* changes in [0.03, 0.20]; (**b**) is for the coefficient *b* in [−1, 2]; (**c**) is for the coefficient *c* from −7 to −2 and (**d**) is for the coefficient *d* from 0.4 to 0.8.

It’s found from the study of fitting function (8) that the coefficients of the fitting function is mainly used to represent the amplitude of the curve above the *x*-axis, so when crossing cortical and cancellous bone, the amplitude above the *x*-axis is small, leading to a small value *a*. Meanwhile, when passing through transitional region, the amplitude above *x*-axis is larger, resulting in a bigger value *a*. The coefficient *b* shows a significant effect on the amplitude ratio of the curve. Term *b* is quite large corresponding to cortical and cancellous bone, while it decreases significantly when it meets with the transitional region. Coefficients *c* and *d* jointly characterize the intersection of the curve with *x*-axis. When drilling through cortical and cancellous bones, the intersection meets the front of the interval, the value of *c* is large and the value of *d* is small. In the transition region, the intersection point is in the middle of the interval where *c* is small and *d* is large. The situation of the coefficients corresponding to different bone layers is shown in Table 2 and changes in shapes of the functions by different coefficients are displayed in Figure 9.

In Table 2, “s” represents small and “h” represents large. It can be seen clearly that the characteristics of the transition region differ a lot from the cortical and cancellous bone, which can be used as the results of the algorithm for bone layer identification.

Figure 9 indicates that coefficient *a* mainly controls the amplitude of the curve and coefficient *b* shows the ratio of the amplitude, while the coefficients *c* and *d* together control the intersection between the curve and the *x*-axis.

**Figure 10:**
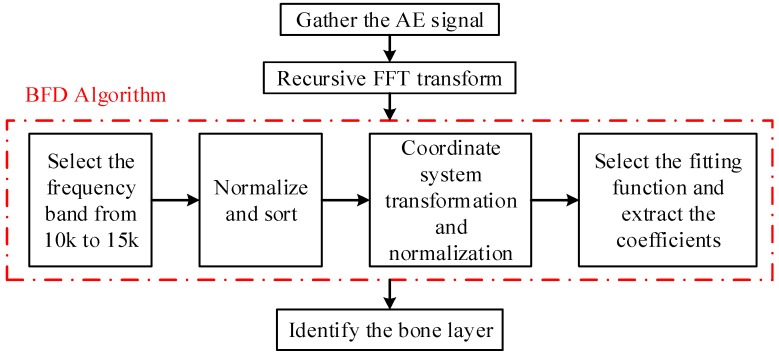
Flow chart of the AE signal process and the proposed FDB algorithm.

To sum up, the whole process can be summarized in several simple steps. Firstly, we collect the AE signals and use the recursive FFT method to transfer the signals into the frequency domain for analysis. Then, we use the proposed FDB algorithm to deal with and get the sets of coefficients as results. Lastly, the bone layer identification can be realized by the coefficients. The whole process can be seen in Figure 10.

### 3.4. Neural Network Model and Parameter Setting

In order to avoid accidental result and prove universal significance of the proposed method, a large amount of experimental data needs to be used to prove it. For the sake of generality, a part of the experimental data can be trained and then the results of the identification will be obtained by testing with other untrained data. The result of recognition rate can be used as an index to evaluate the effectiveness of the algorithm. Among many methods, neural network is a better choice.

**Figure 11:**
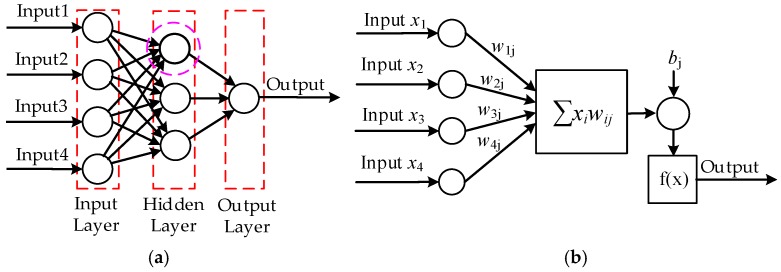
The diagram of the neural network, (**a**) Structure of BP neural network (**b**) Structure of the *j*-th neurons in the hidden layer.

When the coefficients of fitting function are taken as inputs and the recognition result of the bone layer is taken as the output, the input layer of network model has four inputs and the output layer only has one output. According to selection rules of network nodes, the error back propagation (BP) neural network with implicit layer containing three neurons is selected. The neural network model used to train and recognize data collected in experiments, is shown in Figure 11.

In Figure 11b, *x*_1_–*x*_4_ are input values of the eigenvectors, *w_ij_* is the weight corresponding to input *x_i_* and *b_j_* is the corresponding threshold or offset value. The weight and threshold value will continuously change according to the error feedback in the network training process to let the network achieve the best conditions. The fitting function coefficients are used as eigenvectors. In order to facilitate the processing and comparison of data, the eigenvectors need to be normalized to make the Euclidean norm equal to one. For the coefficients *a*, *b*, *c*, *d*, we use the maximum-minimum normalization (9) to process the signals:

(9) $$x\prime =\frac{x-\min x}{\max x-\min x}$$

where *x* is replaced by the coefficients of the fitting function, respectively. The obtained coefficients are distributed in the interval [0, 1].

The transfer functions are selected as follows: the log-sigmoid-type transfer function “logsig”, whose output is between 0 and 1, is selected in hidden layer and the linear transfer function “purelin”, whose input and output values can take any value, is selected in the output layer. However, since the output of the hidden layer is the input of the output layer, the output value of the network is between 0 and 1. The “trainlm” function, which represents the “Levenberg–Marquardt” method and has the fastest training speed in the middle scale of a feed-forward network, is chosen as training function. We set the number training times of the network at 2000, and 10^−6^ as the convergence error and 0.001 as the learning rate. The neural network model can meet the requirements.

In addition, according to the collected force signal, the fitting coefficients at different times after treatment are marked differently. The time when layers are characterized as cortical or cancellous bone by force signal and the coefficients is marked as 0 while the other moments are marked as 1 for transition regions. Due to the fact a linear transfer function is used in output layer, the output value must be distributed between [0, 1] instead of only 0 and 1 values. In order to have a good measure of training and recognition results, we select a function to set the values between 0 and 0.1 to 0 and set the values between 0.9 and 1 to 1.

## 4. Experiments and Result Analysis

### 4.1. Materials and Laboratory Equipment

**Figure 12:**
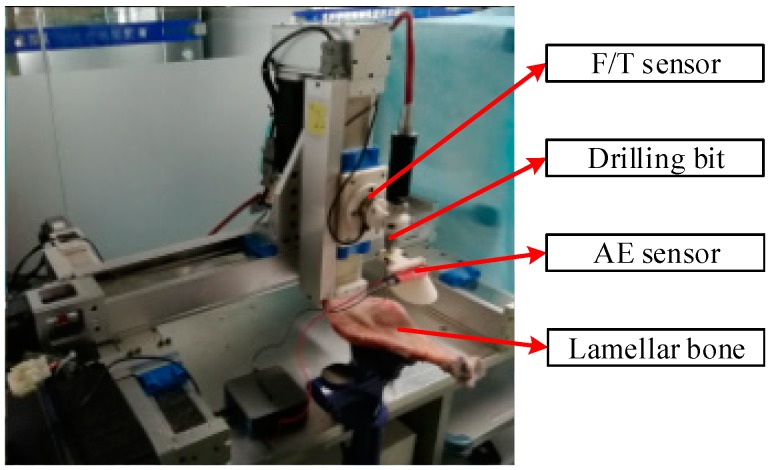
Experimental setup for the drilling process, the drilling bit is used to drill the lamellar bone when the experiment starts, the AE sensor is used to collect the original AE signal and the F/T sensor is used to collect the force and moment signals during the drilling process.

In order to prove the validity of the proposed algorithm and realize the identification of key points, several experiments on different bone samples were carried out. An existing three-axis Cartesian robot is used and the drilling bit is mounted at the end of the robot for drilling operations. A 6-DOF force/moment sensor is also mounted to measure and record thrust force during drilling operations. In addition, AE signals are captured by an AE detector sensor and the collected data is transmitted to a PC through a data acquisition card, just as shown in Figure 12. The maximum sampling rate of the acquisition card is 50 kS/s, which can fully meet the conditions of AE signal acquisition. The lamella used in the experiments are different fresh bones captured from different samples, which have the same bone structure and similar physical and mechanical properties like human bones. The typical structures of bones have cortical and cancellous bone layer, and the trabecular bone, which is an extension of cortical bone in cancellous bone, can be considered as the transition region. In all experiments, a twist drill with a diameter of 2.5 mm is chosen. The speed of the drill bit is 20,000 rpm and the feed rate of the robot end is 1 mm/s. 

The AE sensor, which is mounted on the end of the base of the drilling bit, is about 5 cm away from the tip of the bit during the whole drilling process. The 3D printed device, which is introduced in Section 2.2, is used to enhance the acquisition of AE signals. In order to achieve the best results, the background noise needs to be suppressed. The sounds made by people, motion of the robot and the noise generated when the motors rotate are the main sources of noise generated during the process. The experiments are carried out in a small glass room without unrelated persons, so the noise factors can be minimized. In addition, at the beginning of the analysis, the range of the chosen frequency is between 10 kHz and 15 kHz, which can’t be reached by normal noise, so the noise is filtered out and the influence of the noise on the final results can be neglected.

### 4.2. Experimental Results and Analysis

**Figure 13:**
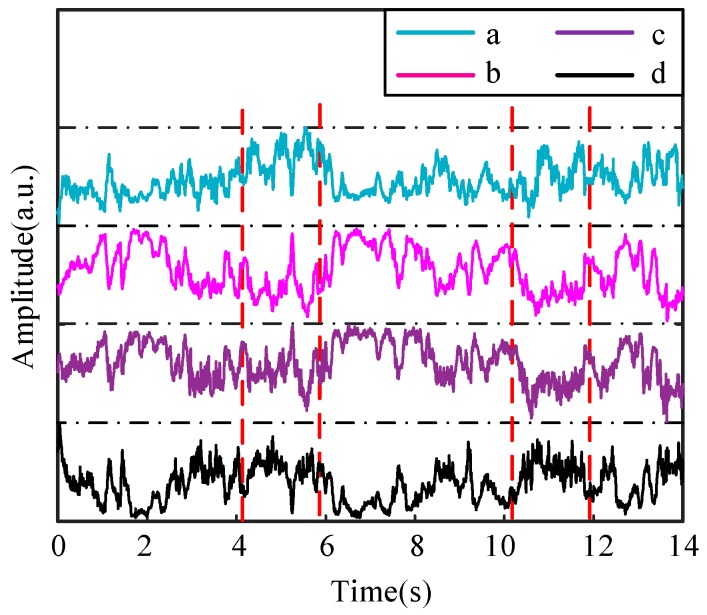
The resulting coefficients after processing by the algorithm, where four kinds of lines represent the different coefficients *a*, *b*, *c* and *d*, respectively. To make the results more clear, the ranges of values are omitted in the figure.

As described in previous section, the experimental data is divided into two groups, one from which is used for training while the other is for testing, and ten sets of data are collected. We select one of the data sets. After using the FDB algorithm for the whole drilling process, we can get four sets of coefficients characteristics *a*, *b*, *c* and *d*. In order to display the trends of the coefficients and realize the bone layer identification by the coefficients, the obtained coefficients are presented in Figure 13.

When the four coefficients suffer a sudden change in value at the same time, according to the analysis introduced above, the bone layer can be considered to have changed. The moment for the sudden change of values, that is, when the bone layer switches from cortical bone to transition region and from transition region to cancellous bone or vice versa, is marked by the red vertical dotted lines in Figure 13. For the reason that the red vertical dot line indicates the changes of the bone layer, the times the lines correspond to can be considered as the boundaries of different bone layers. Times obtained by the lines are shown in Table 3 for comparison.

**Figure 14:**
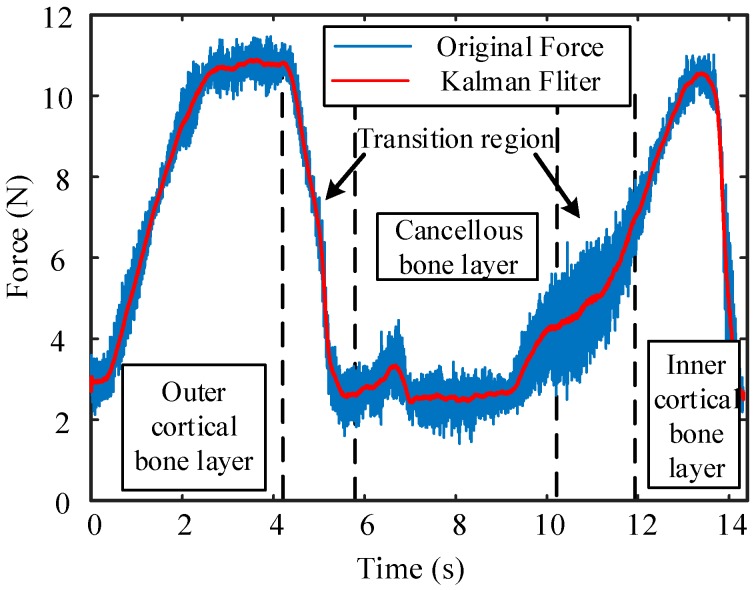
Force signal for bone layer identification. The blue line represents the original data and the red line is the signal after processing by the Kalman filter, which is smoother. Different bone layers for different times are listed in the figure.

In addition, during the drilling process, the F/T sensor collects force signals for analysis. To verify the effectiveness of the proposed method, the force signals are subjected to a Kalman filter and the results are displayed in Figure 14.

Since the signal is selected at the moment when the bit touches the cortical bone, the signal at time 0 corresponds to the cortical bone being drilled. The results obtained by the above methods are shown in Table 3 for comparison and analysis.

The bone drilling feed rate is 0.5 mm/s, so the result of the proposed FDB algorithm corresponds to the data obtained from the force signal. For the reason that we can hardly distinguish the small differences between the transition region visually, and the threshold values of the force signal between two different layers are hard to determine very precisely, the results obtained by direct measurement and the force signals may not be as precise as expected. In contrast, the bone layers determined by the proposed method are more accurate and detailed. The experimental results show that at the moments 4.11 s–5.85 s and 10.18 s–11.9 s, which correspond to the transition region, the values of coefficients *a* and *d* are larger and those of *b* and *c* are smaller than in the other bone layers. The conclusions given by the coefficients, which are obtained by the FDB algorithm, coincide exactly with those of the above theoretical analysis.

### 4.3. Network Training and Result Analysis

Firstly, the fitting coefficients of the ten marked data sets are taken as input values, and the mark is used as output value to the neural network for training. Due to the uneven thickness of the selected lamellar bone, the times required to penetrate the bone are slightly different. The data volume of each group varies slightly from 600 to 800, so the amount of data in the training set is sufficient. After obtaining the trained network, the second group of twenty sets of data are taken as input and tested with the trained neural network. The obtained output result is compared with the marked value to obtain the recognition rates of the sets of data. For the reason that training set does not intersect with the test set and each test set is tested separately, the results obtained have a certain credibility. The recognition rates of the twenty sets of data are shown in Table 4, where it can be seen that the recognition rates are distributed between 75% and 84.2%. For the reason that internal structure of the bone layer is not composed of a single substance and the sound signal acquisition will be influenced by the upper bone media as the depth of the bone increases, the characteristic coefficients of these moments will be affected to varying degrees. To some extent, this influence may lead to wrong identification. Overall, this recognition rates are still quite satisfactory, which also proves the effectiveness of the proposed algorithm.

## 5. Conclusions

In this paper, a bone layer recognition method based on AE signals for orthopedic robots is proposed. In this method, the AE signal collected during the drilling process is processed by FFT, and the status of the bone drilling is characterized by the features obtained by the FDB algorithm. From the result data obtained by this algorithm, the algorithm can distinguish the transitional region between two layers of cortical and cancellous bone and the features are clear and intuitive. Compared with force signals, which are intuitive and consistent with the characterization of bone layers, this algorithm can provide a more elaborate description of the transitional region. The feature quantity obtained from the algorithm and values of marking bone layer according to the corresponding force signal are used as training set to train the neural network, and another twenty sets of irrelevant data are selected for testing. The recognition accuracy reaches 84.2%, indicating that the algorithm has a good recognition effect. This research method highlights the superiority of AE signals in the recognition of bone layers and the sensitivity advantage of this method is unmatched by force signals. The next step of our research will mainly focus on real-time control of drilling robots. The main content involves applying the proposed algorithm to the real-time control of robot systems and verifying the effectiveness and accuracy of the method with more experiments.

## Figures and Tables

**Table 1:** The evaluation for the fitting functions.

SSE	R-Square	RMSE
0.0632 ± 0.0546	0.94795 ± 0.9757	0.0287 ± 0.0164

**Table 2:** The coefficients for different bone layers.

	Coefficients	*a*	*b*	*c*	*d*
Bone Layers					
Cortical bone layer		s	h	h	s
Cancellous bone layer		s	h	h	s
Transition region		h	s	s	h

**Table 3:** Data of the three methods used for the bone layer identification.

	Bone Layer	Cortical Bone	Cancellous Bone	Transition Region
Methods				
By FDB algorithm		0–4.11 s 11.9–14 s	5.85–10.18 s	4.11–5.85 s 10.18–11.9 s
By force signal		0–4.2 s 12.1–14 s	5.9–10.2 s	4.2–5.9 s 10.2–12.1 s

**Table 4:** The recognition rate of the test data.

Data Set	1	2	3	4	5
Recognition rate	81.2%	83.4%	79.1%	75.0%	78.2%
Data set	6	7	8	9	10
Recognition rate	83.3%	84.0%	82.2%	79.9%	77.5%
Data set	11	12	13	14	15
Recognition rate	83.2%	84.2%	82.5%	79.6%	78.8%
Data set	16	17	18	19	20
Recognition rate	82.1%	82.9%	78.4%	79.2%	80.8%

## References

[1] Huang D., Hao D., He B., Wu Q., Liu T., Wang X., Guo H., Fang X. (2015). Posterior atlantoaxial fixation: A review of all techniques. Spine J..

[2] Dai Y., Xue Y., Zhang J. (2014). Noncontact vibration measurement based thoracic spine condition monitoring during pedicle drilling. IEEE/ASME Trans. Mechatron..

[3] Kasai Y., Inaba T., Kato T., Matsumura Y., Akeda K., Uchida A. (2010). Biomechanical study of the lumbar spine using a unilateral pedicle screw fixation system. J. Clin. Neurosci..

[4] Ambati D.V., Wright E.K., Lehman R.A., Kang D.G., Wagner S.C., Dmitriev A.E. (2015). Bilateral pedicle screw fixation provides superior biomechanical stability in transforaminal lumbar interbody fusion: A finite element study. Spine J..

[5] Amirouche F., Solitro G.F., Magnan B.P. (2016). Stability and Spine Pedicle Screws Fixation Strength—A Comparative Study of Bone Density and Insertion Angle. Spine Deform..

[6] Bandiera S., Ghermandi R., Gasbarrini A., Brodano G.B., Colangeli S., Boriani S. (2013). Navigation-assisted surgery for tumors of the spine. Eur. Spine J..

[7] Jarvers J.-S., Katscher S., Franck A., Glasmacher S., Schmidt C., Blattert T., Josten C. (2011). 3D-based navigation in posterior stabilisations of the cervical and thoracic spine: Problems and benefits. Results of 451 screws. Eur. J. Trauma Emerg. Surg..

[8] Fu T.S., Wong C.B., Tsai T.T., Liang Y.C., Chen L.H., Chen W.J. (2008). Pedicle screw insertion: Computed tomography versus fluoroscopic image guidance. Int. Orthop..

[9] Osa T., Abawi C.F., Sugita N., Chikuda H., Sugita S., Tanaka T., Oshima H., Moro T., Tanaka S., Mitsuishi M. (2015). Hand-Held bone cutting tool with autonomous penetration detection for spinal surgery. IEEE/ASME Trans. Mechatron..

[10] Lee W.Y., Shih C.L., Lee S.T. (2004). Force control and breakthrough detection of a bone-drilling system. IEEE/ASME Trans. Mechatron..

[11] Hu Y., Jin H., Zhang L., Zhang P., Zhang J. (2014). State recognition of pedicle drilling with force sensing in a robotic spinal surgical system. IEEE/ASME Trans. Mechatron..

[12] Aziz M.H., Ayub M.A., Jaafar R. (2012). Real-time Algorithm for Detection of Breakthrough Bone Drilling. Procedia Eng..

[13] Kaburlasos V.G., Petridis V., Brett P.N., Baker D.A. (1999). Estimation of the stapes-bone thickness in the stapedotomy surgical procedure using a machine-learning technique. IEEE Trans. Inf. Technol. Biomed..

[14] Federspil P.A., Geisthoff U.W., Henrich D., Plinkert P.K. (2003). Development of the first force-controlled robot for otoneurosurgery. Laryngoscope.

[15] Kim W.Y., Ko S.Y., Park J.O., Park S. (2016). 6-DOF force feedback control of robot-assisted bone fracture reduction system using double F/T sensors and adjustable admittances to protect bones against damage. Mechatronics.

[16] Accini F., Díaz I., Gil J.J. (2016). Using an admittance algorithm for bone drilling procedures. Comput. Methods Programs Biomed..

[17] Dai Y., Xue Y., Zhang J. (2016). A continuous wavelet transform approach for harmonic parameters estimation in the presence of impulsive noise. J. Sound Vib..

[18] Karimi N.Z., Minak G., Kianfar P. (2015). Analysis of damage mechanisms in drilling of composite materials by acoustic emission. Compos. Struct..

[19] Cuadra J., Vanniamparambil P.A., Servansky D., Bartoli I., Kontsos A. (2015). Acoustic emission source modeling using a data-driven approach. J. Sound Vib..

[20] Ubhayaratne I., Pereira M.P., Xiang Y., Rolfe B.F. (2017). Audio signal analysis for tool wear monitoring in sheet metal stamping. Mech. Syst. Signal Process..

[21] Pechenin V.A., Khaimovich A.I., Kondratiev A.I., Bolotov M.A. (2017). Method of controlling cutting tool wear based on signal analysis of acoustic emission for milling. Dyn. Vibroacoustics Mach..

[22] Broda D., Staszewski W.J., Martowicz A., Uhl T., Silberschmidt V.V. (2014). Modelling of nonlinear crack–wave interactions for damage detection based on ultrasound—A review. J. Sound Vib..

[23] Boesnach I., Hahn M., Moldenhauer J., Beth T.H. (2004). Analysis of drill sound in spine surgery. Perspective in Image Guided Surgery.

[24] Pohl B.M., Jungmann I.O., Christ O., Hofmann U.G. Automated drill-stop by SVM classified audible signals. Proceedings of the 2012 Annual International Conference of the IEEE Engineering in Medicine and Biology Society (EMBC).

[25] Sun Y., Jin H., Hu Y., Zhang P., Zhang J. State recognition of bone drilling with audio signal in robotic orthopedics surgery system. Proceedings of the IEEE/RSJ International Conference on Intelligent Robots and Systems.

[26] Liao Z., Axinte D.A. (2016). On monitoring chip formation, penetration depth and cutting malfunctions in bone micro-drilling via acoustic emission. J. Mater. Process. Technol..

[27] Santiuste C., Rodríguez-Millán M., Giner E., Miguélez H. (2014). The influence of anisotropy in numerical modeling of orthogonal cutting of cortical bone. Compos. Struct..

[28] Sezek S., Aksakal B., Karaca F. (2012). Influence of drill parameters on bone temperature and necrosis: A FEM modelling and in vitro experiments. Comput. Mater. Sci..

